# Sustained Intraocular Pressure Control After iStent Infinite^®^ Implantation for Steroid-Induced Glaucoma: A Case Report

**DOI:** 10.3390/jcm15041658

**Published:** 2026-02-22

**Authors:** Kyunghee Lee, Je Hyun Seo, Leslie Jay Katz, Alex S. Huang, Su-Ho Lim

**Affiliations:** 1 Division of Nephrology, Hypertension and Kidney Transplantation, University of California, Irvine, CA 92868, USA; gmldufdf@gmail.com; 2 Division of Nephrology, Internal Medicine, Daegu Veterans Health Service Medical Center, Daegu 42835, Republic of Korea; 3 Veterans Medical Research Institute, Veterans Health Service Medical Center, Seoul 05368, Republic of Korea; jazmin2@naver.com; 4 Wills Eye Hospital, Philadelphia, PA 19107, USA; ljkatz@willseye.org; 5 Hamilton Glaucoma Center, Shiley Eye Institute, Viterbi Family Department of Ophthalmology, University of California San Diego, San Diego, CA 92037, USA; aahuang@health.ucsd.edu; 6 Department of Ophthalmology, Daegu Veterans Health Service Medical Center, Daegu 42835, Republic of Korea

**Keywords:** glaucoma, steroid-induced glaucoma, minimally invasive glaucoma surgery, iStent infinite, trabecular meshwork

## Abstract

**Background/Objectives:** Steroid-induced glaucoma (SIG) or ocular hypertension is a well-known complication after corticosteroid exposure to the eye, particularly intravitreal dexamethasone implantation. The main mechanism of elevated intraocular pressure (IOP) is trabecular meshwork dysfunction, leading to increased aqueous outflow resistance. Although most SIG cases respond to medical treatment, some patients develop persistent IOP elevation, requiring surgical intervention. Minimally invasive glaucoma surgery (MIGS) has recently emerged as a safer surgical option, but there are a limited number of reports using MIGS for SIG. **Methods**: A 73-year-old man, who had branch retinal vein occlusion with refractory macular edema despite multiple anti-VEGF injections, received an intravitreal Ozurdex^®^ (Allergan, Irvine, CA, USA) implant. He developed marked IOP elevation from 17 to 34 mmHg despite maximal topical therapy. Visual field progression and progressive retinal nerve fiber layer thinning were also observed. Given the need for continued ocular steroid use and only having one arm due to trauma making drops difficult, three trabecular micro-bypass stent devices (iStent infinite^®^, Glaukos Corp., Aliso Viejo, CA, USA) were implanted for IOP control. Postoperatively, IOP decreased to 13 mmHg and remained stable at 15 mmHg for 12 months. Additionally, macular edema was well-controlled with ongoing Ozurdex treatment and no observed IOP spikes. **Conclusions**: This is the first reported case of SIG-associated Ozurdex successfully managed with triple trabecular micro-bypass stents. The iStent infinite implantation provided safe and sustained IOP control for SIG, highlighting its potential role in patients requiring continuous intravitreal steroids.

## 1. Introduction

Steroid-induced glaucoma (SIG) and ocular hypertension are well-documented complications of corticosteroid therapy, caused by increased resistance to aqueous humor outflow through the trabecular meshwork (TM) [1,2]. Underlying mechanisms include the accumulation of extracellular matrix materials, cytoskeletal reorganization, inhibition of cellular phagocytic activity, and altered myocilin and fibronectin expression in TM cells [1,2,3,4]. Clinically significant intraocular pressure (IOP) elevation can be caused by steroid use of various durations and application routes, including topical, periorbital, intravitreal, or systemic corticosteroid administration. In particular, the sustained-release dexamethasone implant (Ozurdex^®^, Allergan, Irvine, CA, USA) has been widely used for macular edema secondary to retinal vein occlusion, but 20–36% of patients experience transient or sustained IOP elevation requiring medical or surgical management [5,6]. Analyses of adverse events reported in the U.S. Food and Drug Administration Adverse Event Reporting System database between 2010 and 2024 indicate that increased intraocular pressure was among the more frequently reported events following dexamethasone implant administration [7].

Management of SIG is often challenging because affected patients may continue to require corticosteroid therapy for underlying ocular or systemic conditions. Conventional filtering surgery is usually effective in lowering IOP but it also carries significant risks, including postoperative hypotony, bleb-related infection, or fibrosis [8]. Recently, minimally invasive glaucoma surgeries (MIGS), including stents, microhooks, ablation, and other approaches, have emerged as safer options that target physiologic aqueous outflow pathways with less surgical trauma [9,10,11,12]. Among these, trabecular bypass devices such as the iStent platform (iStent, iStent inject, iStent inject W, iStent Infinite, Glaukos Corp., Aliso Viejo, CA, USA) [9,10,11,12,13,14] or the Hydrus Microstent (Alcon, Fort Worth, TX, USA) [9,12] directly create microbypass channels through the TM into Schlemm’s canal, addressing the primary site of steroid-induced outflow resistance [15].

Currently, there are few reports about the application of MIGS, including iStent [13,14], canaloplasty [16], and gonioscopy-assisted transluminal trabeculotomy with or without goniotomy [17,18], or minimally invasive bleb surgery (MIBS) including XEN gel stent (Allergan Inc, Abbvie Company, Irvine, CA, USA) or PreserFlo MicroShunt (Santen Pharmaceutical, Osaka, Japan) [19,20,21] for SIG. Recently, Xiao and Qiu reported that minimally invasive glaucoma surgeries (MIGS) using goniotomy and GATT were utilized in approximately 4.1% of cases as part of a multimodal management approach for steroid-induced ocular hypertension [22]. However, to the best of our knowledge, there were no reports using iStent Infinite for Ozurdex-associated SIG. Thus, we report a case of Ozurdex-associated SIG successfully treated using iStent infinite implantation, achieving long-term IOP control with sustained macular edema resolution despite continued intravitreal steroid injection.

## 2. Case Presentation

**Figure 1:**
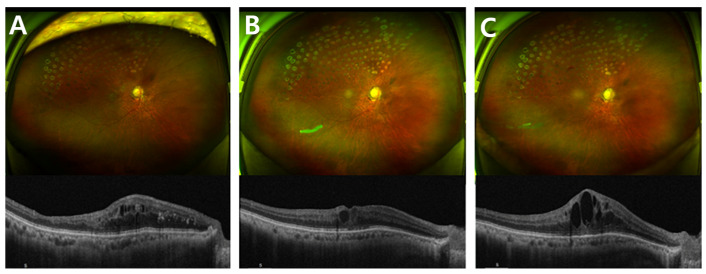
Fundus photograph and OCT changes showing the course of macular edema before and after intravitreal therapy. Wide-field fundus photographs and corresponding spectral-domain optical coherence tomography (SD-OCT) scans demonstrate the sequential changes in macular edema associated with branch retinal vein occlusion (BRVO). (**A**) Prior to intravitreal steroid injection, diffuse cystoid macular edema persisted despite multiple anti-VEGF treatments (bevacizumab and aflibercept). (**B**) One week after intravitreal dexamethasone implant (Ozurdex^®^), a marked reduction in macular edema and improvement in foveal contour were observed. (**C**) At three months post-injection, recurrent cystoid changes and retinal thickening were noted, consistent with the waning effect of the dexamethasone implant.

The study was conducted in accordance with the Declaration of Helsinki, and approved by the Institutional Review Board of Daegu Veterans Hospital (2025-35 and 1 December 2025), and informed consent was obtained from patient involved in the study. A 73-year-old man with a history of kidney transplantation and systemic immunosuppression (tacrolimus and mycophenolate mofetil) presented with persistent macular edema secondary to branch retinal vein occlusion (BRVO) in his right eye. Despite multiple intravitreal anti-vascular endothelial growth factor (anti-VEGF) injections, including bevacizumab and aflibercept, macular edema remained refractory. Consequently, intravitreal dexamethasone implant was administered (Figure 1).

Prior to receiving the Ozurdex implant, his IOP in the right eye had remained well-controlled at 17 mmHg on topical dorzolamide/timolol fixed combination (DTFC, Cosopt^®^, Santen Pharmaceutical Co., Ltd., Osaka, Japan) twice daily due to ocular hypertension. The left eye showed IOP with 15–17 mmHg without topical glaucoma medication. The central corneal thickness was 542 μm in the right eye and 548 μm in the left eye. Baseline gonioscopy revealed wide-open angles (Spaeth grade D40r in all quadrants, both eyes) with moderate trabecular pigmentation (3+ in the right eye and 2+ in the left eye). The cup-to-disk ratio was 0.6 in the right eye and 0.4 in the left eye. More specifically, the patient had ocular hypertension in his right eye, with IOP of 24–26 mmHg before DTFC treatment. Although peripheral visual field defect was present, the visual field defect was clinically attributed to prior laser treatment for BRVO rather than to glaucomatous optic neuropathy. Accordingly, the patient was considered to have ocular hypertension rather than established glaucoma before steroid exposure.

Within several months after implantation, the patient developed progressive ocular hypertension, with IOP rising to 26 mmHg despite triple topical therapy with DTFC, brimonidine (Alphagan^®^, Allergan, Abbvie company, Irvine, CA, USA), and bimatoprost (Lumigan^®^, Abbvie company, Irvine, CA, USA). The IOP subsequently increased further to 34 mmHg, and Humphrey 24-2 visual field testing revealed rapid stepwise deterioration (mean deviation, −7.56 dB, −10.20 dB, and −21.91 dB; visual field index, 87%, 82%, and 36% respectively). All visual field tests met the standard reliability criteria, with false-positive and false-negative rates < 15%, and fixation loss < 20%. And a confirmatory visual field test one day before surgery demonstrated nearly identical results, supporting true progression.

**Figure 2:**
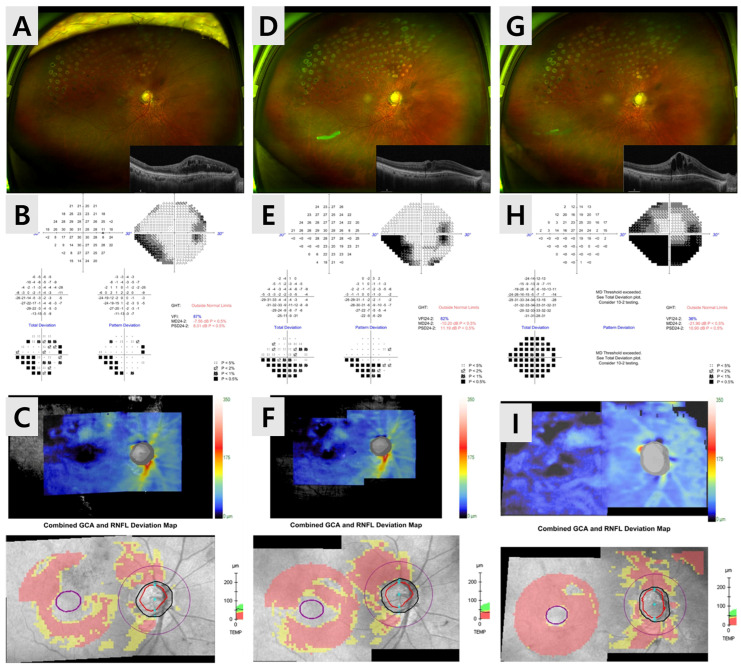
Progressive functional and structural deterioration following intravitreal Ozurdex implantation. (**A**–**C**) Baseline wide-field fundus photograph, OCT, and Humphrey 24-2 visual field show moderate field loss (MD = −7.56 dB; VFI = 87%). (**D**–**F**) One month after Ozurdex^®^, despite partial reduction in macular edema, IOP increased to 26 mmHg, and the visual field further deteriorated (MD = −10.20 dB; VFI = 82%), accompanied by RNFL and GCA thinning on deviation maps. (**G**–**I**) At three months, IOP rose to 34 mmHg with marked worsening of visual field (MD = −21.91 dB; VFI = 36%) and diffuse loss of RNFL and GCA signal, consistent with steroid-induced glaucomatous progression.

Fundus photography demonstrated a hyperemic optic disk and diffuse laser scars from prior focal photocoagulation. Optical coherence tomography (OCT) revealed progressive cystoid macular edema and structural thinning of the retinal nerve fiber layer (RNFL) and ganglion cell analysis, particularly in the superior and inferior arcuate regions, consistent with rapid glaucomatous progression (Figure 2).

The patient had also lost one of his upper limbs due to previous trauma, making the self-administration of multiple topical medications difficult. Given his fragile systemic condition and difficulty using frequent topical eye drops, conventional filtration surgery or tube surgery were considered highly risky, and therefore, we opted for a minimally invasive approach targeting the trabecular outflow pathways.

Slit lamp examination revealed fine corneal opacities, a properly placed posterior chamber intraocular lens, and absence of iris neovascularization. Gonioscopy revealed a wide-open angle with the absence of peripheral anterior synechiae or neovascularization of the angle.

**Figure 3:**
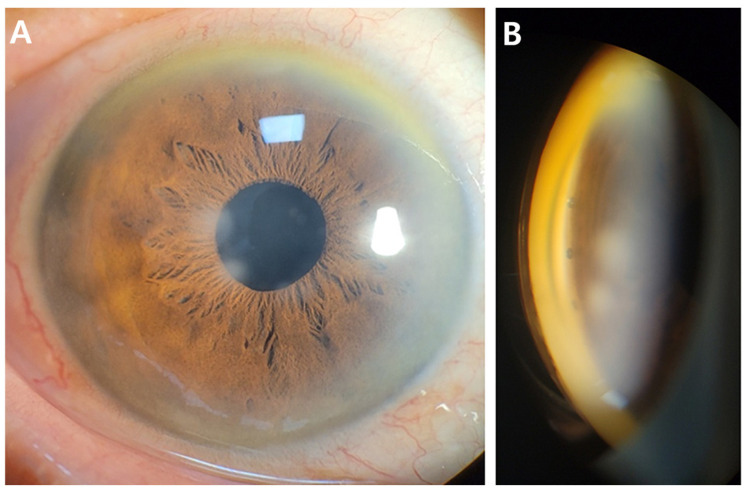
Postoperative one month slit lamp photographs (**A**) and gonioscopic findings (**B**). The three stents are clearly visible within the pigmented trabecular meshwork on indirect gonioscopy. The anterior chamber is quiet, with neither inflammation, peripheral anterior synechiae, nor stent obstruction.

**Figure 4:**
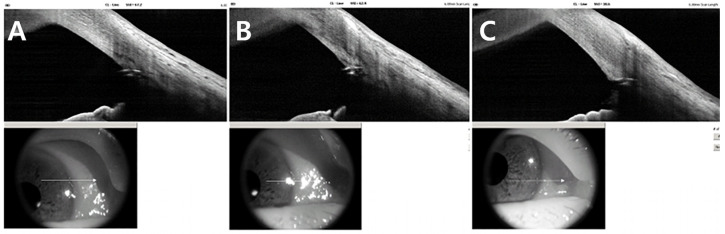
Postoperative anterior segment optical coherence tomography (AS-OCT) for the iStent infinite position on 1 month. AS-OCT scans show three iStent infinite trabecular microbypass stents placed along the nasal quadrant (**A**–**C**). (**C**) The lowest stent appears slightly over-implanted, with its proximal edge minimally recessed into the trabecular plane; however, the device maintains full contact with Schlemm’s canal and is likely functional. The reference infrared reference images from the lower panels indicate scan locations across each stent.

Based on these findings, the mechanism of IOP increase was assumed to be due to steroid-induced TM dysfunction with increased trabecular outflow resistance. To re-establish physiologic aqueous outflow via the Schlemm’s canal, an iStent infinite implantation was planned. The procedure was performed under topical anesthesia using a clear corneal incision. Three trabecular micro-bypass stents were implanted sequentially into the nasal quadrant under direct gonioscopic visualization. Postoperatively, good positioning of all three stents within the pigmented TM without significant inflammation or hyphema was observed on slit lamp examination (Figure 3). AS-OCT demonstrated similar findings except for one slightly over-implanted stent in the inferonasal side (Figure 4).

**Figure 5:**
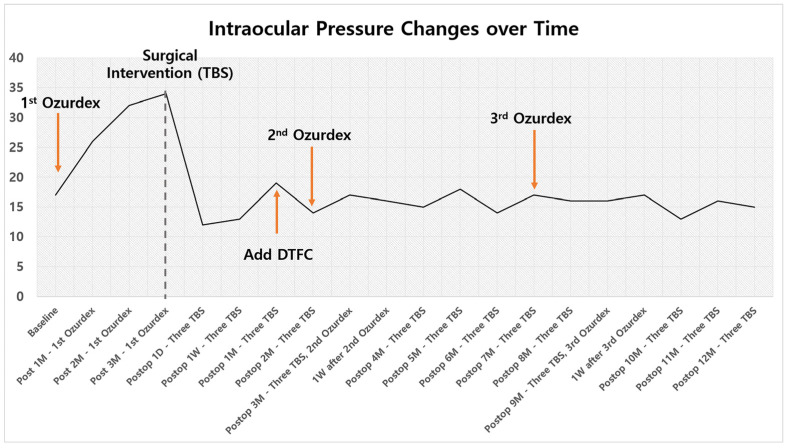
Intraocular pressure changes over time. Abbreviations; TBS = Trabecular microbypass stent; M = Month; D = Day; W = Week; DTFC = Dorzolamide/Timolol Fixed Combination.

**Figure 6:**
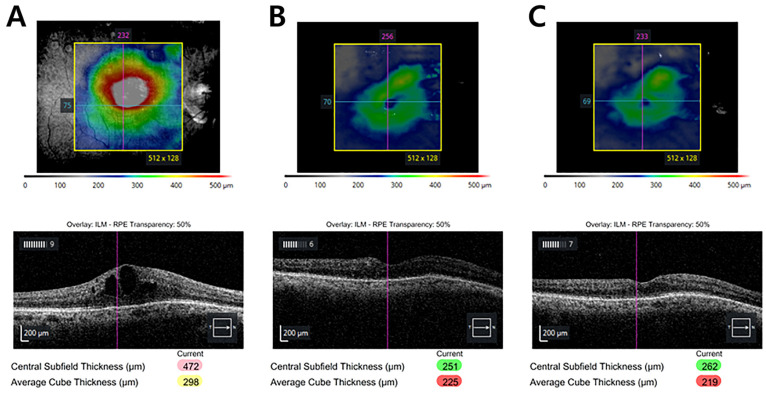
Longitudinal changes in optical coherence tomography (OCT) demonstrating resolution and stabilization of macular edema. (**A**) Preoperative OCT showing severe cystoid macular edema with a central subfield thickness of 472 μm. (**B**) At 14 weeks after three trabecular microbypass stents implantation, following the second Ozurdex injection administered at week 12, the macular contour improved significantly with a central subfield thickness of 251 μm. (**C**) At 12 months after three trabecular microbypass stents implantation, corresponding to approximately 3 months after the third Ozurdex injection, the foveal profile remained stable (262 μm) with no recurrence of intraretinal fluid, consistent with anatomical recovery and sustained macular edema control.

The postoperative course was uneventful. IOP decreased to 13 mmHg on postoperative day 7, remaining stable at 15 mmHg with DTFC at the last follow-up at 12 months. Table 1 and Figure 5 summarized the changes in IOP. There were neither intraoperative nor postoperative complications. Notably, the patient underwent two additional intravitreal Ozurdex injections for recurrent BRVO-related macular edema, which remained well-controlled, as confirmed by serial macular OCT images showing resolution of cystoid changes and restoration of foveal contour (Figure 6).

To date, the patient has maintained excellent IOP control and visual stability for over one year following three trabecular micro-bypass stent surgeries, despite long-standing exposure to intravitreal corticosteroids. This case demonstrates that utilizing trabecular micro-bypass MIGS may be effective in restoring trabecular outflow and maintaining long-term pressure control in SIG, even when further steroid therapy is clinically necessary.

## 3. Discussion

In this case study, a patient exhibited marked IOP elevation with secondary SIG and underwent iStent infinite implantation along the nasal quadrant for IOP control. IOP improved from a maximum preoperative IOP of 34 on maximal medical treatment to 13 mmHg at postoperative 1 week. IOP control was maintained through 12 months (15 mmHg) on only one fixed combination eyedrop. Furthermore, additional Ozurdex implantations were continued with no recurrence of IOP elevation. These results suggest that multiple trabecular micro-bypass stents could achieve relatively long-term IOP control even in the presence of dexamethasone implant exposure.

In this patient, pre-existing ocular hypertension should be carefully considered, as individuals with ocular hypertension or established glaucomatous disease are at higher risk of corticosteroid-induced IOP elevation (“steroid response”) and subsequent steroid-induced glaucoma.

Management of SIG is thought to be highly complex or sometimes challenging, particularly if cessation of steroids is difficult [1,2]. Usually, IOP rise most commonly occurs between three to six weeks and normalizes within two weeks after cessation of steroid therapy [2]. A previous study [23] compared intravitreal and periocular triamcinolone for macular edema from BRVO. Interestingly, the incidence of an IOP rise of 20 mm Hg or greater was significantly higher in the intravitreal group (33.3%), compared to the retrobulbar group (7.4%) [23]. The problem is that most ocular pathologies that require intravitreal corticosteroids need multiple injections to maintain therapeutic effect, leading to increased risk of IOP spikes also [1,4]. In this context, sustained-release corticosteroid implants have an important role in the treatment of various ocular microvascular or inflammatory pathologies. Ozurdex is the shortest-acting and biodegradable implant, which releases dexamethasone at a controlled rate for up to six months [5,6]. Regarding the incidence of IOP elevation, about one-third of patients in each implant group (34.1% for the 0.35 mg group and 36.0% for the 0.7 mg group) had a clinically significant IOP increase, necessitating treatment in the MEAD study, a randomized clinical trial of varying doses of dexamethasone implant [5].

Initial SIG management typically involves topical antiglaucoma medication, including aqueous beta-blockers, carbonic anhydrase inhibitors, alpha-agonists, or prostaglandin analogs. However, in some cases, particularly those with severe steroid response or for those patients who require prolonged exposure, laser trabeculoplasty or surgical intervention is needed. Conventional filtration surgery or tube shunt implantation can achieve substantial IOP reduction but carry notable risks, including hypotony, bleb infections, and fibrosis [8]. To prevent severe complications, previous studies reported on goniotomy, gonioscopy-assisted transluminal trabeculotomy, and canaloplasty [16,17,18].

Recently, MIGS have emerged as promising alternatives to traditional filtration surgeries for selected patients with mild to moderate open-angle glaucoma. Xiao and Qui reported Ozurdex-induced ocular hypertension developed within one year of Ozurdex implantation (23.3%, 40 implant/171 implants) [22]. In their study, angle-based MIGS were utilized in seven (4.1%) cases and showed favorable results [22]. Angle-based MIGS procedures target physiological outflow pathways with faster recovery, less tissue disruption, and fewer severe postoperative complications. Major MIGS or micro-invasive bleb surgeries (MIBS) techniques could be classified into four categories: (1) trabecular bypass stents (iStent or Hydrus) [9,10,11,12,13,14], (2) trabecular ablation or excision (Kahook Dual Blade, microhook; New World Medical Inc, Rancho Cucamonga, CA, USA, Trabectome; MicroSurgical Technology, Redmond, WA, USA, or bent angle needle goniotomy) [11,22,24], (3) Schlemm’s canal dilation (canaloplasty) [16], and (4) subconjunctival microstents, MIBS (XEN gel stent and PreserFlo MicroShunt) [19,20,21]. The selection of a specific MIGS/MIBS procedure should be guided by the presumed site of main outflow resistance and the patient’s clinical profile.

SIG is a form of secondary open-angle glaucoma, which aqueous outflow resistance increased [1,2,4]. The primary main pathology is localized to the TM, where there are physical and mechanical changes in the microstructure of the TM, inhibition of TM phagocytosis, and depositions of various substances in the TM [1,2,4]. Therefore, a direct angle-based MIGS procedure may be the better rational approach compared to other approaches. Rationales include: (1) canaloplasty or viscodilation may be less effective in SIG because Schlemm’s canal and the collector channels are often structurally intact, and TM resistance may be increased if corticosteroid therapy continues; (2) subconjunctival MIBS might be aggressive in such cases because Schlemm’s canal and distal channels remain structurally intact; and (3) trabecular ablation procedures may trigger additional fibrosis. In these contexts, we chose to use the iStent infinite stent, which creates permanent channels connecting the anterior chamber to Schlemm’s canal, thus restoring physiologic drainage independent of TM resistance [9,10]. The Hydrus implant was not available in South Korea at the time this patient was cared for. Also, the recently published INTEGRITY Study—a randomized, double-masked, multicenter, 24-month study—reported a statistically significant greater proportion of eyes achieving unmedicated mean diurnal IOP reduction ≥ 20% from baseline in eyes with no surgical complications in the iStent infinite group compared to the Hydrus group [9]. Although we adopted this rationale for choosing iStent infinite over other angle-based procedures, the argument remains speculative. Thus, physicians might consider the other MIGS might have good surgical results, given this is a single-case report. This report represents a single-case experience, and that no generalizable conclusions regarding the relative efficacy of angle-based procedures can be drawn.

The favorable result in our case may be attributed to the additive effect of three trabecular micro-bypass stents, which provides access to up to 240° of Schlemm’s canal. The iStent infinite composed of three heparin-coated titanium stents, allowing for a broader aqueous access [9]. This might be especially beneficial in eyes with localized or segmental outflow obstruction, such as those seen in steroid-induced TM fibrosis or high TM resistance conditions.

In a similar manner, Louca and Wechner reported that two second-generation iStent inject devices may have prevented SIG by creating a trabecular bypass pathway, serving as a long-term, multidirectional safeguard that maintained aqueous humor outflow in a patient with Behçet disease during four years of follow-up [14]. Buchacra et al. also reported four SIG cases treated with a first-generation iStent, with IOP decreasing over 12 months of follow-up (21–30 mmHg before iStent implantation vs. 17–20 mmHg at postoperative 12-month afterward) [25].

Our case report has several limitations inherent to its retrospective single-case report. First, the findings were based on a single patient without a comparator or an alternative angle-based surgical attempt. Second, the duration of follow-up remains limited, relative to the potential need for lifelong or repeated steroid exposure. Third, although the postoperative IOP control was stable, the relative contributions of surgical intervention versus prior or concurrent medical therapy could not be fully disentangled. Accordingly, these findings should be interpreted as hypothesis-generating rather than generalizable. And we acknowledge that macular edema can adversely affect visual field performance and OCT ganglion cell analysis, potentially exaggerating the apparent severity of the functional loss. However, in this case, the pattern of visual field deterioration was considered clinically consistent with glaucomatous progression, rather than a nonspecific macular artifact.

While this single case cannot establish definitive efficacy, it raises the potential for angle-based MIGS to be an effective, safe, and physiologically compatible treatment for ocular hypertension in SIG. Future studies with larger cohorts receiving MIGS for SIG are warranted to confirm long-term safety, durability of pressure reduction, long-term compatibility with intravitreal steroid implants, and utility of AS-OCT for postoperative follow-up.

## 4. Conclusions

To our knowledge, this is the first report of (1) SIG managed with three trabecular micro-bypass stents using the iStent infinite platform, and (2) achieving sustained IOP control and stable macular outcomes over 12 months of follow-up despite continued and repeated intravitreal Ozurdex implantations. This case demonstrates successful long-term control of SIG with the iStent infinite trabecular micro-bypass system in a patient requiring continued intravitreal corticosteroid injection for macular edema. The favorable outcome suggests that trabecular bypass MIGS can effectively restore aqueous outflow in steroid-related trabecular dysfunction while minimizing surgical risk in patients who are elderly or who are compromised due to systemic co-morbidities.

## Figures and Tables

**Table 1:** Changes in IOP over time.

Timeline	IOP (mmHg)	Medication	Intervention
Baseline	17	DTFC	□
After 1 Mo—1st Ozurdex	26		
After 2 Mo—1st Ozurdex	32	Add Bimatoprost	
After 3 Mo—1st Ozurdex	34	Add Brimonidine	
**Glaucoma Surgery (Three Trabecular Microbypass Stents, iStent Infinite)**			
Postop 1 D	12	No	
Postop 1 W	13	No	
Postop 1 Mo	19	Restart DTFC	
Postop 2 Mo	14	DTFC	
Postop 3 Mo (2nd Ozurdex)	17	DTFC	2nd Ozurdex
1 W after 2nd Ozurdex	16	DTFC	
Postop 4 Mo	15	DTFC	
Postop 5 Mo	18	DTFC	
Postop 6 Mo	14	DTFC	
Postop 7 Mo	17	DTFC	
Postop 8 Mo	16	DTFC	
Postop 9 Mo (3rd Ozurdex)	16	DTFC	3rd Ozurdex
1 W after 3rd Ozurdex	17	DTFC	
Postop 10 Mo	13	DTFC	
Postop 11 Mo	16	DTFC	
Postop 12 Mo	15	DTFC	□

Mo = Month; D = Day; W = Week; DTFC = Dorzolamide/Timolol Fixed Combination.

## Data Availability

The original contributions presented in this study are included in the article. Further inquiries can be directed to the corresponding author (Su-Ho Lim).

## Abbreviations

The following abbreviations are used in this manuscript:

IOP	Intraocular pressure
MIGS	Minimally invasive glaucoma surgery
MIBS	Minimally invasive bleb surgery
TM	Trabecular meshwork
DTFC	Dorzolamide-Timolol Fixed Combination
BRVO	Branch Retinal Vein Occlusion
OCT	Optical coherence tomography
AS-OCT	Anterior segment optical coherence tomography
RNFL	Retinal nerve fiber layer

## References

[1] Razeghinejad M.R., Katz L.J. (2012). Steroid-induced iatrogenic glaucoma. Ophthalmic Res..

[2] Weinreb R.N., Polansky J.R., Kramer S.G., Baxter J.D. (1985). Acute effects of dexamethasone on intraocular pressure in glaucoma. Invest. Ophthalmol. Vis. Sci..

[3] Lu R., Kolarzyk A.M., Stamer W.D., Lee E. (2025). Human ocular fluid outflow on-chip reveals trabecular meshwork-mediated Schlemm’s canal endothelial dysfunction in steroid-induced glaucoma. Nat. Cardiovasc. Res..

[4] Patel P.D., Kodati B., Clark A.F. (2023). Role of Glucocorticoids and Glucocorticoid Receptors in Glaucoma Pathogenesis. Cells.

[5] Boyer D.S., Yoon Y.H., Belfort R., Bandello F., Maturi R.K., Augustin A.J., Li X.Y., Cui H., Hashad Y., Whitcup S.M. (2014). Three-year, randomized, sham-controlled trial of dexamethasone intravitreal implant in patients with diabetic macular edema. Ophthalmology.

[6] Liu D., Chen Y., Fu X., Zhao Y., Ji L., Qiu Y., Li S. (2025). The relationship between the intraocular position of dexamethasone intravitreal implant and post-injection intraocular pressure elevation. Front. Med..

[7] Zhao C.F., Lan L., Shi X.Y., Li J., Fan S. (2025). Assessment the real-world safety of intravitreal dexamethasone implant (Ozurdex): Novel insights from a comprehensive pharmacovigilance analysis utilizing the FAERS database. BMC Pharmacol. Toxicol..

[8] Gedde S.J., Herndon L.W., Brandt J.D., Budenz D.L., Feuer W.J., Schiffman J.C., Tube Versus Trabeculectomy Study Group (2012). Postoperative complications in the Tube Versus Trabeculectomy (TVT) study during five years of follow-up. Am. J. Ophthalmol..

[9] Ahmed I.I.K., Berdahl J.P., Yadgarov A., Reiss G.R., Sarkisian S.R., Gagne S., Robles M., Voskanyan L.A., Sadruddin O., Parizadeh D. (2025). Six-Month Outcomes from a Prospective, Randomized Study of iStent infinite Versus Hydrus in Open-Angle Glaucoma: The INTEGRITY Study. Ophthalmol. Ther..

[10] Kim M., Rho S., Lim S.H. (2023). Five-Year Outcomes of Single Trabecular Microbypass Stent (iStent((R))) Implantation with Phacoemulsification in Korean Patients. Ophthalmol. Ther..

[11] Mori S., Tanito M., Shoji N., Yokoyama Y., Kameda T., Shoji T., Mizoue S., Saito Y., Ishida K., Ueda T. (2022). Noninferiority of Microhook to Trabectome: Trabectome versus Ab Interno Microhook Trabeculotomy Comparative Study (Tram Trac Study). Ophthalmol. Glaucoma.

[12] Tan J.C.K., Clement C., Healey P., Lim R., White A., Yuen J., Agar A., Lawlor M. (2025). Long-term comparative outcomes of Hydrus versus iStent inject microinvasive glaucoma surgery implants combined with cataract surgery. Br. J. Ophthalmol..

[13] Guemes-Villahoz N., Garcia-Feijoo J., Martinez-de-la-Casa J.M., Torres-Imaz R., Donate-Lopez J. (2021). Trabecular microbypass stent to treat ocular hypertension after intravitreal injection of a dexamethasone implant. J. Fr. Ophtalmol..

[14] Louca M., Wechsler D.Z. (2025). Protection from Steroid-Induced Glaucoma via iStent Inject in a Patient with Behcet’s Disease. Ophthalmol. Glaucoma.

[15] Hohberger B., Haug M., Bergua A., Lammer R. (2020). MIGS-off-label option for treatment-refractory steroid-induced ocular hypertension. Ophthalmologe.

[16] Brusini P., Tosoni C., Zeppieri M. (2018). Canaloplasty in Corticosteroid-Induced Glaucoma. Preliminary Results. J. Clin. Med..

[17] Chen R.I., Purgert R., Eisengart J. (2023). Gonioscopy-Assisted Transluminal Trabeculotomy and Goniotomy, With or Without Concomitant Cataract Extraction, in Steroid-Induced and Uveitic Glaucoma: 24-Month Outcomes. J. Glaucoma.

[18] Ucgul R.K., Olgun A.O., Akdeniz Z.B., Inan P., Aktas Z., Ucgul A.Y. (2025). Gonioscopy-assisted transluminal trabeculotomy for intractable ocular hypertension after repeated intravitreal dexamethasone implant injections. Int. Ophthalmol..

[19] Rezkallah A., Mathis T., Denis P., Kodjikian L. (2019). XEN Gel Stent to Treat Intraocular Hypertension After Dexamethasone-Implant Intravitreal Injections: 5 Cases. J. Glaucoma.

[20] Tan S.Y., Md Din N., Mohd Khialdin S., Wan Abdul Halim W.H., Tang S.F. (2021). Ab-Externo Implantation of XEN Gel Stent for Refractory Steroid-Induced Glaucoma After Lamellar Keratoplasty. Cureus.

[21] Bourauel L., Petrak M., Holz F.G., Mercieca K., Weber C. (2025). Short-Term Safety and Efficacy of PreserFlo Microshunt in Patients with Refractory Intraocular Pressure Elevation After Dexamethasone Implant Intravitreal Injection. J. Clin. Med..

[22] Xiao J., Qiu M. (2025). Management of ocular hypertension following intravitreal dexamethasone implant (ozurdex). Am. J. Ophthalmol. Case Rep..

[23] Hayashi K., Hayashi H. (2005). Intravitreal versus retrobulbar injections of triamcinolone for macular edema associated with branch retinal vein occlusion. Am. J. Ophthalmol..

[24] Epstein R., Taravella M., Pantcheva M.B. (2020). Kahook Dual Blade goniotomy in post penetrating keratoplasty steroid-induced ocular hypertension. Am. J. Ophthalmol. Case Rep..

[25] Buchacra O., Duch S., Milla E., Stirbu O. (2011). One-year analysis of the iStent trabecular microbypass in secondary glaucoma. Clin. Ophthalmol..

